# Augmented particle trapping and attenuated inflammation in the liver by protective vaccination against *Plasmodium chabaudi *malaria

**DOI:** 10.1186/1475-2875-8-54

**Published:** 2009-04-02

**Authors:** Jürgen Krücken, Denis Delić, Heike Pauen, Anna Wojtalla, Manal El-Khadragy, Mohamed A Dkhil, Horst Mossmann, Frank Wunderlich

**Affiliations:** 1 Institute for Parasitology, University of Veterinary Medicine Foundation, Bünteweg 17, Hannover, Germany; 2 Division of Molecular Parasitology and Centre for Biological and Medical Research, Heinrich-Heine-University Düsseldorf, Düsseldorf, Germany; 3 Zoology Department, College of Science, King Saud University, Saudi Arabia; 4 Max Planck Institute for Immunobiology, Freiburg, Germany

## Abstract

**Background:**

To date all efforts to develop a malaria vaccine have failed, reflecting the still fragmentary knowledge about protective mechanisms against malaria. In order to evaluate if vaccination changes responses of the anti-malaria effectors spleen and liver to blood stage malaria, BALB/c mice succumbing to infection with *Plasmodium chabaudi *were compared to those surviving after vaccination.

**Methods:**

Mice were vaccinated with host cell plasma membranes isolated from *P. chabaudi*-infected erythrocytes. Hepatic and splenic capacity to trap particulate material was determined after injection of fluorescent polystyrol beads. Hepatic gene expression was measured using real-time RT-PCR and Northern blotting.

**Results:**

Survival of BALB/c mice was raised from 0% to 80% and peak parasitaemia was decreased by about 30% by vaccination. Vaccination boosted particle trapping capacity of the liver during crisis when splenic trapping is minimal due to spleen 'closing'. It also attenuated malaria-induced inflammation, thus diminishing severe damages and hence liver failure. Vaccination increased hepatic IFN-γ production but mitigated acute phase response. Vaccination has a complex influence on infection-induced changes in expression of hepatic nuclear receptors (CAR, FXR, RXR, and PXR) and of the metabolic enzymes Sult2a and Cyp7a1. Although vaccination decreased CAR mRNA levels and prevented Cyp7a1 suppression by the CAR ligand 1,2-bis [2-(3,5-dichloropyridyloxy)]benzene (TCPOBOP) on day 8 p.i., Sult2a-induction by TCPOBOP was restored.

**Conclusion:**

These data support the view that the liver is an essential effector site for a vaccine against blood stage malaria: vaccination attenuates malaria-induced inflammation thus improving hepatic metabolic activity and particle trapping activity of the liver.

## Background

Despite intense efforts to develop a vaccine against malaria during the last 30 years, a safe and effective vaccine candidate is not yet available [1]. One reason for this failure may be that knowledge about the effector sites and mechanisms that have to be activated for successful protection is still rather incomplete. Moreover, natural immunity to malaria underlies rather complex control. It is directed against the blood stages of *Plasmodium *parasites, but it is never solid, i.e. it mitigates and can even completely abolish disease symptoms, but it does not prevent re-infections during malaria seasons [2,3]. In an experimental malaria model, *Plasmodium chabaudi *in rodents, a vaccination model has been previously developed that resembles natural immunity in so far that it helps susceptible mice to survive an otherwise lethal blood stage infection without preventing parasitaemia [4]. This vaccination model uses host cell plasma membranes of *P. chabaudi*-parasitized red blood cells (pRBC) as an immunogen. These erythrocyte membranes contain several parasite proteins [5,6] the functions of which have remained unknown to date, including a major immunogenic *P. chabaudi *protein *Pc*90 against which most of the antibodies induced by this type of vaccination are directed [4]. This vaccination model is used here to further study the effector sites and mechanisms, which have to be activated to survive blood stage infections.

The spleen is widely considered to be the central effector site of the host defence against blood stage malaria [7,8], and it is thought to destroy pRBC by the same mechanisms which normally remove senescent and other aberrant erythrocytes from circulation [9]. Basically, pRBC are eliminated by macrophages in the red pulp areas of the spleen, specifically in extravascular beds through which blood is percolated before reaching the collecting veins. This open circulation and, hence, the direct contact between pRBC and macrophages, has been described to become 'closed' during acute *P. chabaudi *malaria or at least transiently closed during acute *Plasmodium yoelii *17XNL [10,11] and *P. chabaudi adami *malaria [12]. However, it is possible that vaccination prevents – at least partially – this 'closing' mechanism, thus enabling the spleen to destroy pRBC during crisis when parasitaemia is dramatically falling.

The liver is another important effector site against blood stage malaria, though research in this field is largely neglected to date [13,14]. Indeed, research concentrates on the role of the liver in the pre-erythrocytic development of parasites. However, the liver, although not exhibiting any extravascular beds as the spleen, is also able to phagocytose senescent erythrocytes [15,16] and pRBC [17,18]. In particular, the intravascular Kupffer cells, which constitute about 80–90% of all resident macrophages of the reticuloendothelial system [19], are competent for erythrophagocytosis. In *P. chabaudi *malaria it has been recently shown that the liver improves its trapping capacity, especially during crisis of self-healing infections, i.e. that phase, when the spleen is 'closed' [13,20]. However, the effect of protective vaccination on liver trapping capacity has never been investigated to date.

Here, protective vaccination against blood stages of *P. chabaudi *is shown to convert non-healer BALB/c mice to self-healer mice. This vaccination-induced self-healing coincides with an augmented trapping capacity of the liver – but not of the spleen – especially during crisis, when parasitaemia drops from more than 50% to about 1% or even below. Furthermore, vaccination boosts production of IFN-γ and strongly attenuates inflammation and promotes recovery of liver metabolism from infection-induced dysregulation during crisis.

## Methods

### Mice

BALB/c mice were bred under specific pathogen-free conditions in the central animal facilities at the Max-Planck-Institute for Immunobiology in Freiburg and at the University of Düsseldorf. Experiments were performed only with female mice at an age of 10–14 weeks. They were housed in plastic cages and received standard diet (Wohrlin, Bad Salzufeln, Germany) and water *ad libitum*. In some experiments, mice received 60 μg of the synthetic CAR ligand 1,2-bis [2-(3,5-dichloropyridyloxy)] benzene (TCPOBOP) in 100 μl DMSO by intraperitoneal injection 24 h before they were killed for isolation of RNA. All experiments were approved by the state authorities and followed German law on animal protection.

### Infections

A non-clonal line of *P. chabaudi *was used which behaves very similarly to *P. chabaudi chabaudi *AS in terms of restriction fragment length polymorphism analysis [14]. Also, the AS clone and the line used here reveal sequence identity for dihydrofolate reductase and for a cysteine protease [21] with only a single nucleotide exchange in the latter (Krücken and Wunderlich, unpublished data). Blood stages of *P. chabaudi *were weekly passaged in NMRI mice [14,22]. BALB/c mice were challenged with 10^6 ^*P. chabaudi*-parasitized erythrocytes by intraperitoneal injection. Parasitaemia was evaluated in Giemsa-stained blood smears. Total erythrocytes were counted in a Neubauer chamber.

### Vaccination

Mice were vaccinated with a modified procedure that has been developed previously [4]. As an immunogen, host cell plasma membranes were used which were isolated in the form of ghosts from *P. chabaudi*-pRBC as described elsewhere [23]. About 10^6 ^ghosts in 100 μl FCA were subcutaneously injected twice, three weeks and one week before challenging with *P. chabaudi *pRBC. Controls received plasma membranes in the form of ghosts from non-infected erythrocytes in FCA or only FCA.

### Flow cytometry

Spleens were processed for flow cytometry in a FACScan (BD Biosience) as detailed previously [14]. After aseptic removal, spleens were gently dissociated through a stainless steel sieve into RPMI medium (Invitrogen) supplemented with 5% foetal calf serum (PAA Laboratories). After lysis of erythrocytes by NH_4_Cl, total leukocytes were counted in a Neubauer chamber. Leukocytes were preincubated with anti-CD16/CD32 (FcIII/II receptor) FC block (BD Bioscience) for 15 min and then labelled with one of the following FITC-labelled monoclonal antibodies: anti-mouse CD45R/B220 (clone RA3-6B2), anti mouse CD4 (H129.19), anti-mouse CD8a (53-6.7), anti-mouse CD244.2 (2B4), anti-mouse Br1 (RB6-8C5) (all BD Bioscience), and anti-mouse F4/80 (C1:A3-1) (Immunokontact). FACS analyses were done with a sample size of 10.000 cells gated on the basis of forward and sideward scatter [24]. Data were stored and processed using Cell Quest Pro software (BD Bioscience).

### Trapping capacity

Trapping of 3 μm green fluorescent polystyrol beads (Ducke Scientific corporation, Palo Alto, California) was measured according to the procedure described recently [13,20]. Particles were intravenously injected, mice were killed 5 min later, spleens and parts of livers were removed, weighed and then incubated in ethanolic KOH, to which 5 × 10^5 ^red fluorescent beads (diameter 2.9 μm) were added as an internal standard. The samples were incubated until complete tissue resolution before fluorescence of beads was measured in a spectrometer (Perkin Elmer LS 55, Germany) at excitation/emission wavelengths (450/480) and (520/590) for green and red beads, respectively. For localization of beads [20], cryosections of spleen and liver were stained with haematoxilin and eosin. Fluorescence and bright field pictures of the same field were take separately and superimposed electronically.

### RNA-Isolation

Spleens and liver pieces were aseptically removed, rapidly frozen, and stored in liquid N_2_. Total RNA was isolated using Trizol (Invitrogen).

### Northern blot analysis

Total RNA (20 μg) was glyoxylated, separated in agarose gels, and transferred to positively charged Biodyne7PLUS nylon membrane (PALL Corporation, Pensacola, FI) as described previously [25]. Hybridiziation was carried out with cDNA fragments labelled with [α^32^P]dCTP using the DecaLabel™ DNA Labeling Kit (Fermentas) in ExpressHyb solution (Clontech) at 65°C overnight [26]. Blots were washed at 65°C twice for 15 min in 2 × SSC, 0.1% SDS and twice for 30 min in 0.1 × SSC, 0.1% SDS, and subjected to autoradiography at -80°C using Biomax MS film and screen (Kodak). For densitometric analyses, autoradiographs were scanned and evaluated using QuantiScan 3.0 software (Biosoft, Cambridge, UK).

### Quantitative real-time RT-PCR

Contaminating genomic DNA was removed from total RNA by digestion with DNase using the DNA-free™ kit (Ambion). Then, cDNA was synthesized using the QuantiTect^® ^Reverse Transcription kit (Qiagen) which includes an additional step to remove contaminating genomic DNA. Amplifications were performed in a TaqMan7500 (AppliedBiosystems) using QuantiTect™ SYBR^® ^Green PCR kit (Qiagen) and gene-specific QuantiTect™ primer assays (Qiagen) according to the manufacturer's instructions. Following an initial incubation at 50°C for 2 min, Taq polymerase was activated by incubation at 95°C for 10 min. During the following 55 cycles made up of 15 s at 95°C, 35 s at 60°C, and 30 s at 72°C, the amount of double stranded PCR product was measured as SYBR green fluorescence at the end of the extension phase. All PCR reactions yielded only a single product species of the expected size as revealed by melting point analysis and gel electrophoresis. Relative quantitative evaluation of amplification data was done using Taqman7500 system software v.1.2.3f2 (AppliedBiosystems) and the 2^-ΔΔc^_T _method [27]. Expression of the genes of interest was compared to 18S rRNA.

### Statistical analyses

Differences between vaccinated and non-vaccinated mice were analysed using Student's t tests.

## Results

### Protective vaccination

**Figure 1:**
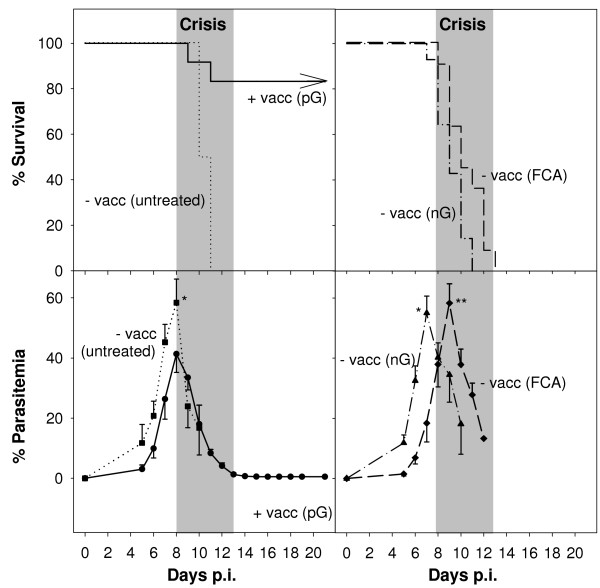
**Vaccination-induced protection against *P. chabaudi *malaria**. BALB/c mice were vaccinated with ghosts from pRBC (+ vacc (pG); n = 18) or were not vaccinated (- vacc (untreated); n = 8) or were treated with ghosts from non-infected erythrocytes in FCA (nG; n = 8), or treated only with FCA (FCA; n = 17), before challenging with 10^6 ^pRBC. All values are means and half standard errors of the mean (S.E.M.). **, p < 0.01, *, p < 0.05 vs. peak parasitaemia in vaccinated mice. The crisis phase (days 8–13 p.i.) is accentuated in grey.

The vaccination procedure used here converts mice from a non-healer to a self-healer phenotype, i.e. it prevents mortality, but it does not prevent parasitaemia [4]. Indeed, female BALB/c mice are highly susceptible to *P. chabaudi *malaria. Challenge with 10^6 ^*P. chabaudi*-parasitized erythrocytes resulted in a peak parasitaemia of approximately 58% on day 7 *p. i*. (Figure 1). Though the following crisis was characterized by falling parasitaemia, all mice succumbed to infection during this period. Vaccination, however, protected BALB/c mice against otherwise fatal *P. chabaudi *malaria. Indeed, more than 80% of these mice survived the infection (Figure 1). Vaccination did not prevent parasitaemia, but peak parasitaemia was significantly decreased to 42%, which dropped during crisis to about 1% and in individual mice to even below 1% on day 13 p.i. (Figure 1). Mice vaccinated with erythrocyte ghosts from non-infected mice were not protected, i.e. all mice succumbed to infection during crisis. The same was observed in control mice, which received only FCA. Remarkably, blood glucose level, anaemia, and percentage of reticulocytes in the blood did not significantly differ between vaccinated and non-vaccinated mice (Additional file 1).

### Particle trapping by liver and spleen

**Figure 2:**
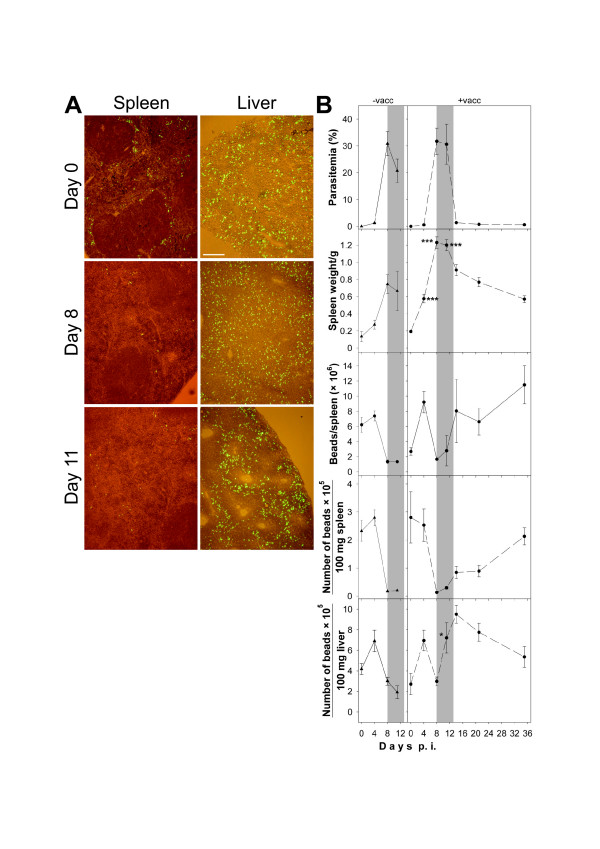
**Splenic and hepatic particle trapping during *P. chabaudi *malaria**. (A) Localization of 3 μm green fluorescent beads in splenic and hepatic cryosections of non-vaccinated BALB/c mice. The scale bar represents 200 μm. (B) Quantification of splenic and hepatic trapping. BALB/c mice were vaccinated (+vacc) or not vaccinated (-vacc) and then were challenged with 10^6 ^pRBC. Parasitaemia, spleen weight, and total number of beads per spleen, and numbers of beads per 100 mg spleen or liver are given as means ± S.E.M. The crisis phase (days 8–13 p.i.) is accentuated in grey. ***, p < 0.001 vs. non-vaccinated control on the same day p.i. *, p < 0.05 vs. non-vaccinated control on the same day p.i.

**Figure 3:**
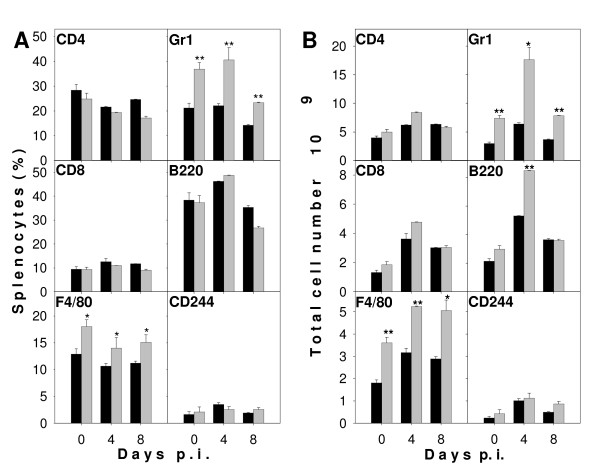
**Cellular composition of the spleen during *P. chabaudi *malaria**. Vaccinated (black columns) and non-vaccinated (gray columns) mice were infected with 10^6 ^*P. chabaudi *pRBC and spleen cells were isolated on the days indicated. The frequency (A) and total number (B) of CD4^+ ^and CD8^+ ^T cells, F4/80^+ ^macrophages, Gr1^+ ^granulocytes, B220^+ ^B cells, and CD244^+ ^NK cells among nucleated spleen cells are given as means + half S.E.M. **, p < 0.01 vs. non-vaccinated control on the same day p.i. *, p < 0.05 vs. non-vaccinated control on the same day p.i.

Though vaccination did not directly affect spleen size in terms of weight, there was an indirect effect on size, which became evident upon infection. Thus, increase in splenic weight of vaccinated mice was about twice as much as in non-vaccinated mice (Figure 2A). In the latter, average spleen weight rose from about 130 mg on day 0 to about 660 mg on day 8 p.i. Infection of vaccinated mice resulted in a dramatic increase to more than 1,200 mg per average spleen on day 8 p.i. (Figure 2B). On day 0 p.i., vaccination already induced a significantly increased proportion (Figure 3A) and absolute number (Figure 3B) of phagocytes in the spleen, i.e. frequency of Gr1^+ ^granulocytes increased by about 75% and that of F4/80^+ ^macrophages increased by about 40% (Figure 3). This vaccination-induced increase in phagocytes was retained during infection. However, the frequency of different lymphoid cell populations was not significantly altered by vaccination with the exception of B220^+ ^B cells showing a significant but temporary increase in absolute number on day 4 p.i. (Figure 3B).

In preliminary experiments, particle trapping by spleen and liver was analysed by fluorescence microscopy after injection of 3 μm fluorescent beads. Splenic cryosections, taken on day 0 p.i., clearly show trapping of beads predominantly in the marginal zone of the spleen that was lost around peak parasitaemia (Figure 2A) as described previously for C57BL/6 mice [20]. Hepatic sections reveal a more or less uniform distribution of trapped beads in the liver on days 0 and 8 p.i., however, on day 11 p.i. beads appear to be restricted predominantly to regions with large distance to the central veins (Figure 2A). The cryosections were insufficient to resolve whether trapped beads have been directly phagocytosed by macrophages, adhere to the surface of phagocytic cells or are passively trapped in the organs. Incidentally, paraffin sectioning was not possible since all solvents used for dewaxing the sections did also dissolve the beads.

Since number of beads varied considerably between slides a more quantitative method employing dissolution of tissues by ethanolic KOH followed by fluorescence spectroscopy was used to look for differences in particle trapping between vaccinated and non-vaccinated mice. Despite the increase in the frequency of phagocytic cells in the spleen, vaccination did not significantly affect the specific capacity of the spleen to trap particles – neither before nor during infection. After injection of 3 μm fluorescent polystyrol beads, approximately 2.2 × 10^5 ^particles/100 mg spleen were trapped in non-vaccinated mice and about the same number in vaccinated mice before infection (Figure 2B). On day 4 p.i., trapping capacity remained at about the same level as before, but at peak parasitaemia on day 8 p.i., there was a dramatic reduction in particle trapping both in vaccinated and non-vaccinated mice (Figure 2B).

Obviously, the entry of particles into the spleen was largely prevented at peak parasitaemia. This so-called 'closing' of the spleen lasted during the major part of crisis and, only at the end of crisis, a slight 'reopening' began in vaccinated mice, but the spleen re-gained its initial trapping capacity only on day 36 *p.i*. – if at all (Figure 2B). Despite the enormous malaria-induced splenomegaly in vaccinated mice, total splenic uptake of fluorescent beads was not significantly higher during crisis. However, splenomegaly resulted in rapid recovery of total splenic trapping capacity after crisis.

Since preliminary tests had shown that there were no significant effects of vaccination or *P. chabaudi *infection on liver weight, trapping capacity was only determined for pieces of liver and no total liver trapping capacity was determined. In contrast to spleen, specific trapping capacity per 100 mg liver was significantly affected by vaccination, which became evident upon infection during crisis (Figure 2B). Indeed, vaccination did neither affect the size of the liver nor modify the capacity of the liver to trap particles before infection. On day 4 p.i., there was an increase in the trapping capacity of the liver, but on day 8 p.i., there was a decline to about the initial trapping capacity with no difference between vaccinated and non-vaccinated mice (Figure 2B). During crisis, particle trapping further dropped in non-vaccinated control mice, whereas vaccinated mice exhibited a sharp increase in their capacity to trap particles in the liver and this increase further progressed reaching a maximum on day 14 p.i., i.e. shortly after crisis (Figure 2B).

### Liver inflammation

**Figure 4:**
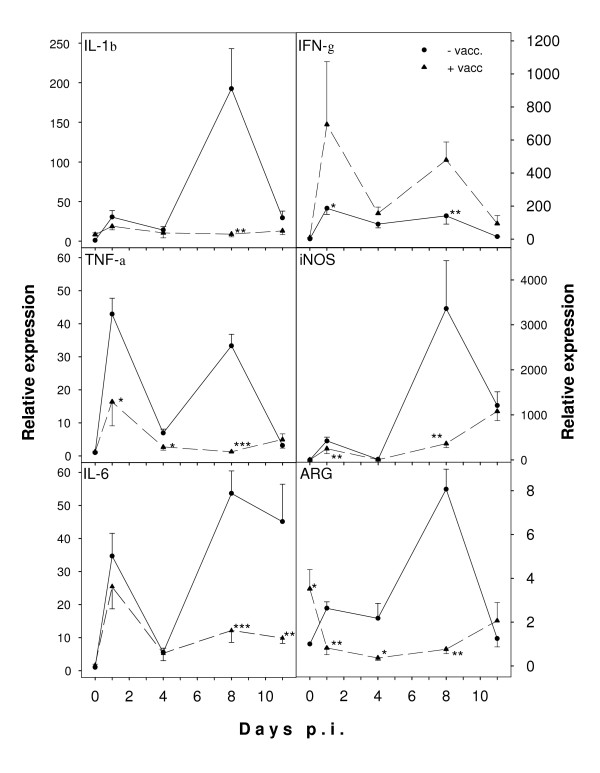
**Real-time RT-PCR analysis of cytokines, iNOS, andarginase (ARG) in the liver**. Expression was analysed in hepatic RNA from vaccinated (circles) and non-vaccinated (triangles) mice on the indicated days after challenging with *P. chabaudi*. Signals for genes of interest were normalized to 18S rRNA signals and relative expression is given as fold increase compared to non-vaccinated mice on day 0 p.i. All values are mean and half S.E.M. ***, p < 0.001 vs. non-vaccinated control on the same day p.i. **, p < 0.01 vs. non-vaccinated control on the same day p.i. *, p < 0.05 vs. non-vaccinated control on the same day p.i.

In order to detect possible effects of vaccination on inflammatory and immune responses in the liver, real-time RT-PCR was used to measure the proinflammatory cytokines IL-1β, TNF, and IL-6 and the T_H_1 cytokine IFN-γ, which is protective against *P. chabaudi *malaria [28-30]. The mRNA levels of these cytokines followed a biphasic pattern in non-vaccinated mice during infection, with a first peak on day 1 p.i. and a second peak on day 8 p.i. (Figure 4). After vaccination, infection induced a large increase in expression of IFN-γ especially on days 1 and 8 p.i., whereas vaccination dampened the infection-induced increases in mRNA levels for the proinflammatory cytokines IL1-β, TNF, and IL-6 – in particular around peak parasitaemia. Kupffer cells are a well-known source of these cytokines in the liver suggesting strong Kupffer cell activation in non-vaccinated mice during malaria. In accordance, mRNA levels of iNOS, marker for M1 macrophages, also exhibited a biphasic expression pattern in response to infection, whereas induction of iNOS by *P. chabaudi *malaria is largely depressed in vaccinated mice (Figure 4). Arginase, a marker for alternatively activated M2 macrophages, followed about the same expression profile as iNOS although at much lower levels.

**Figure 5:**
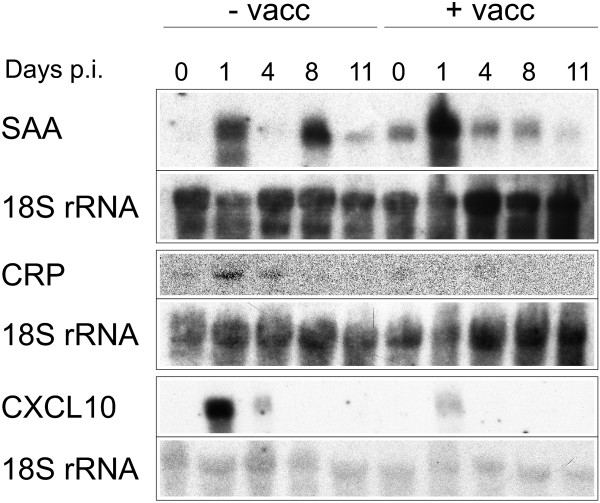
**Northern blot analyses of inflammatory markers in the liver**. Hepatic RNA isolated from *P. chabaudi*-infected vaccinated (+ vacc) or non-vaccinated (-vacc) mice on the days indicated was hybridized to probes recognizing all isoforms of SAA, CRP, or CXCLl10 before reprobing with an 18S rRNA-specific probe. Blots are representative of at least three independent experiments.

In the liver, the above proinflammatory cytokines are especially known to induce acute phase and other innate immune responses. Expression profile of two acute phase proteins and one proinflammatory chemoattractant were, therefore, compared between vaccinated and non-vaccinated mice. Similar to the cytokines, total serum amyloid A (SAA) followed a biphasic kinetic during infection in non-vaccinated mice (Figure 5). Vaccination, however, abolished this biphasic pattern due to decreased expression, especially on days 8 and 11 p.i. By contrast, C-reactive protein (CRP) and the CXC chemokine ligand CXCL10 were activated only in the early phase of precrisis, i.e. on days 1 and 4 p.i., and their induction by infection was largely prevented by vaccination.

### Liver metabolism

**Figure 6:**
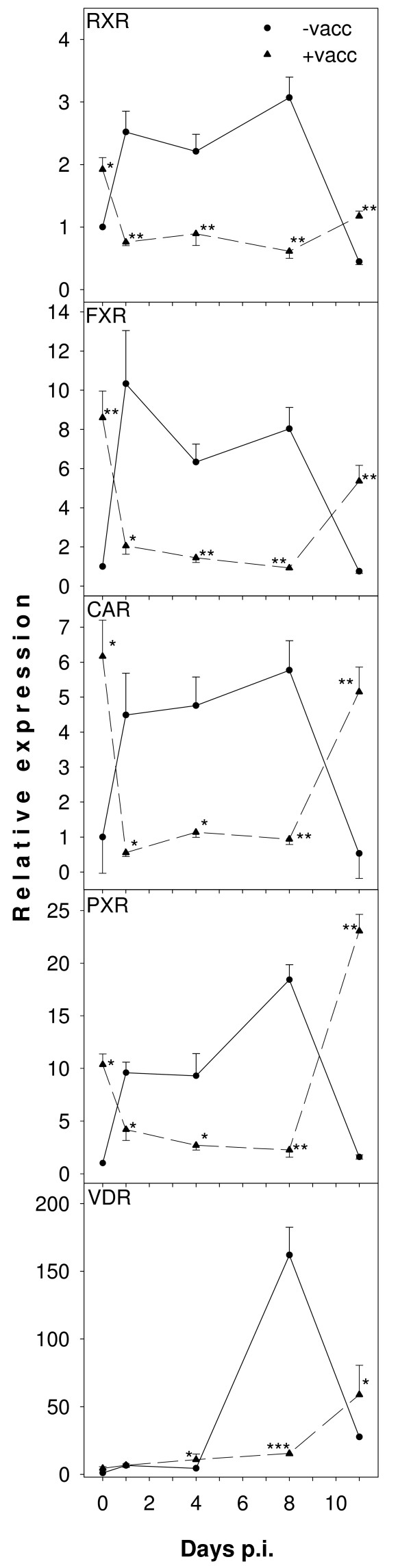
**Real-time RT-PCR analyses of nuclear receptor expression in the liver**. Levels of RXR, FXR, CAR, PXR, and VDR mRNA were analysed in hepatic RNA of vaccinated (circles) and non-vaccinated (triangles) mice on the indicated days after *P. chabaudi *infection. Signals were normalized to 18S rRNA and relative expression is given as fold increase compared to non-vaccinated mice on day 0 p.i. All values are mean and half S.E.M. **, p < 0.01 vs. non-vaccinated control on the same day p.i. *, p < 0.05 vs. non-vaccinated control on the same day p.i.

IL-1β, TNF and IL-6 are also well known to depress liver metabolism by interfering with expression of key nuclear receptors regulating expression of hepatocyte-specific metabolic enzymes [31-35]. Therefore, mRNA levels of five members of the nuclear receptor family were examined by real-time RT-PCR. Infection caused a transiently increased expression of RXR, FXR, CAR, PXR, and VDR in non-vaccinated mice, however, expression dramatically dropped during crisis on day 11 p.i (Figure 6). In contrast, vaccinated mice initially showed increased mRNA levels of these receptors before infection (day 0 p.i.), whereas infection down-regulated their expression on days 1, 4 and 8 p.i. (Figure 6). Surprisingly, however, these receptors were significantly up-regulated again during crisis. By the end of crisis, there was an almost complete recovery of RXR, FXR, and CAR to those levels induced by vaccination before infection, whereas PXR and VDR were significantly higher expressed (Figure 6).

**Figure 7:**
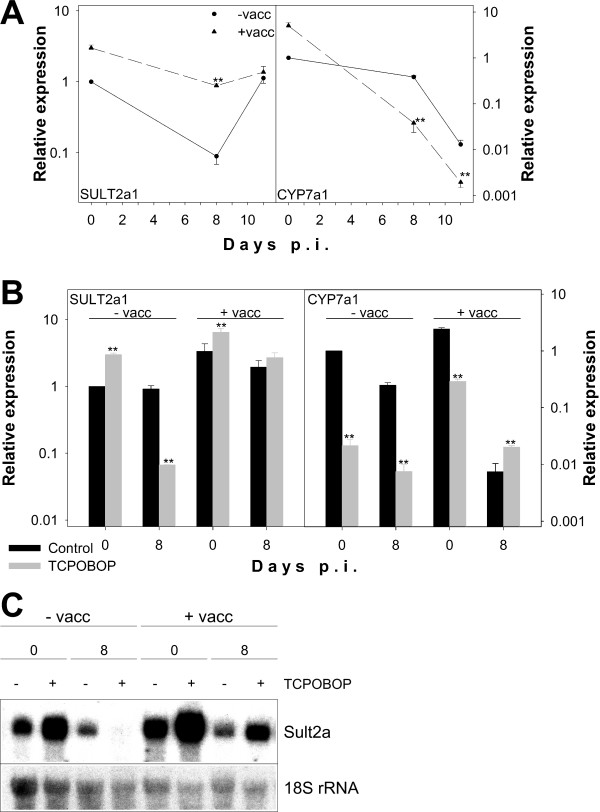
**Effects of vaccination on malaria-induced suppression of SULT2a and CYP7a1**. (A) Analysis of hepatic SULT2a1 and CYP7a1 expression on the indicated days of *P. chabaudi *malaria in vaccinated (triangles) and non-vaccinated (circles) mice by real-time RT-PCR. Signals were normalized to 18S rRNA signals and relative expression is given as fold increase compared to non-vaccinated mice on day 0 p.i. All values are means and half S.E.M. (B) Analysis of TCPOBOP effects on expression of SULT2a1 and CYP7a1. Vaccinated (+ vacc) or non-vaccinated (- vacc) mice were subjected to injection of TCPOBOP (grey columns) or vehicle (black columns) before analysis of SULT2a1 and CYP7a1 expression on the indicated days of *P. chabaudi *infection using real-time RT-PCR as in (A). (C) TCPOBOP effects on expression of SULT2a were confirmed by Northern blotting. Blot is a representative of three independent experiments. Equal loading of the blot was confirmed by rehybridization with a probe specific for 18S rRNA.

Rapid destruction of pRBC is expected to lead to highly elevated levels of cholesterol-derived toxic bile acids and haem-derived bilirubin. Since CAR is involved in regulation of both bile acid detoxification [36] and bilirubin elimination [37], CAR activity during malaria is of particular interest. Expression of the positive and negative CAR response genes Sult2a and Cyp7a1 involved in bile acid detoxification and cholesterol degradation to bile acids, respectively, were measured by real-time RT-PCR. These analyses confirmed suppression of Sult2a mRNA levels by blood stage malaria in BALB/c mice on days 8 and 11 p.i. (Figure 7A) as previously shown in C57BL/6 and LTβR^-/- ^mice [13,38]. Vaccination, however, increased Sult2a1 mRNA levels by approximately two- and tenfold on days 0 and 8 p.i., respectively (Figure 7A). Cyp7a1 was severely down-regulated on days 8 and 11 p.i., whereas vaccination already induced expression on day 0 p.i. In contrast to Sult2a1, however, infection strongly intensified Cyp7a1 down-regulation in vaccinated mice (Figure 7A).

In order to test directly, whether elevated CAR mRNA levels on day 8 p.i. correspond to higher hepatic receptor activity, mice were challenged with TCPOBOP, a nuclear receptor ligand known to up-regulate Sult2a [39] and to down-regulate Cyp7a1 [40] via activation of CAR. Figure 7B clearly shows that treatment with TCPOBOP approximately doubles Sult2a1 mRNA levels in both vaccinated and non-vaccinated mice on day 0 p.i. Surprisingly, non-vaccinated mice on day 8 p.i. did not show any TCPOBOP-inducibility, but rather displayed TCPOBOP-induced suppression of Sult2a1 levels. This suppression is not observable in vaccinated mice, which maintained inducibility of Sult2a1 by TCPOBOP (Figure 7B), although they exhibited lower expression of the TCPOBOP receptor CAR (Figure 6). In order to substantiate these very unusual results, these experiments were not only confirmed three times with new groups of mice but also using Northern blotting (Figure 7C) to exclude any errors due to quantification by PCR.

TCPOBOP-regulation of Cyp7a1 expression revealed an exactly reciprocal effect. As expected, TCPOBOP suppressed expression of Cyp7a1 in non-vaccinated mice on days 0 and 8 p.i. and in vaccinated mice on day 0 p.i. (Figure 7B). In contrast, however, Cyp7a1 mRNA levels were elevated in vaccinated mice on day 8 p.i. after treatment with TCPOBOP. Therefore, negative regulation of Cyp7a1 was restricted to non-vaccinated mice with high CAR mRNA levels, while positive regulation of Sult2a by TCPOBOP was only observable in vaccinated mice with low CAR mRNA levels.

## Discussion

The vaccination model under investigation here protects mice against blood stages of *P. chabaudi *malaria. It does not prevent parasitaemia, though peak parasitaemia is significantly decreased, but rather helps mice to overcome the disease. Indeed, vaccination converts non-healer to self-healer mice [4]. Self-healing of *P. chabaudi *malaria in turn is known to be associated with acquiring long-lasting protective immunity against homologous re-challenge [41]. This capability is normally controlled by genes of the *H-2 *complex and genes of the non-*H-2 *background [42-44]. The susceptible BALB/c mice exhibit the malaria-'resistant' *H-2*^*d *^haplotype, but a malaria-'susceptible' non-*H-2 *background [44]. Hence, the vaccination procedure used here abolishes/overcomes those genetic restrictions which cause susceptibility and which are controlled by mouse non-*H-2 *genes.

Vaccination of BALB/c mice appears to improve the potential phagocytic capacity of the spleen as indicated by a significant increase in the percent proportion of both G1^+ ^granulocytes and F4/80^+ ^macrophages as well as the concomitant increase in spleen size. Nevertheless, this did not contribute to an increased trapping capacity since the spleen became almost 'closed' during peak parasitaemia and subsequent crisis, when masses of pRBC were destroyed as evidenced by dramatically falling parasitaemias [25]. In contrast to spleen, however, trapping capacity of the liver is augmented after vaccination, especially during crisis. This augmentation is preceded by a remarkable increase in the *P. chabaudi*-induced production of IFN-γ-mRNA. In accordance, IFN-γ is known to activate the host defence in other malaria vaccination models too [45,46]. This supports the view that the liver can function as an active effector against malarial blood stages, in particular during crisis, when the spleen is 'closed' [20], i.e. when the spleen excludes the uptake of pRBC and, thus, cannot be mechanically involved in the drop of peripheral parasitaemia observed during crisis.

In the liver, the Kupffer cells are important sites for phagocytosis of damaged and senescent erythrocytes [15,16], and phagocytic activity of Kupffer cells during *Plasmodium berghei *malaria has been described to be increased both in vitro [18] and in perfused livers [17]. The vaccination procedure used here is shown to modulate hepatic inflammation as indicated by altered responses of typical macrophage activation parameters towards to *P. chabaudi *malaria. Indeed, *P. chabaudi *infections induce dramatic up-regulations of IL-1β, TNF, IL-6, and iNOS in the non-vaccinated susceptible BALB/c mice. High levels of TNF have been shown to result in lethal hepatic damage during blood stage *P. chabaudi *malaria [47]. Indeed, the increases in TNF and IL-6 are even about 10-times higher than during lethal endotoxic shock [48]. Also, iNOS expression observed at peak parasitaemia is comparable to that expression occurring during LPS-induced lethal shock [49]. These high levels of proinflammatory cytokines and especially iNOS appear to contribute to severe liver failure, which entails lower particle trapping during crisis. In the vaccination model used here, however, there is a significant dampening of the infection-induced increase in IL-1β, TNF, IL-6 and iNOS, especially observable at peak parasitaemia. This indicates that vaccination does not prevent activation of Kupffer cells and other inflammatory cells by malaria infection but rather prevents their over-activation and, hence, severe liver failure. In this context, it is also noteworthy that high levels of IL-1β and TNF prevent efficient phagocytosis by Kupffer cells, reasonably due to impaired hepatic microvascular blood flow by promoting leukocyte adhesion to the walls of sinusoids [50]. Thus, vaccination-induced decreases in the expression of these cytokines, together with the decreased production of the chemoattractant CXCL10, presumably allow improved blood circulation in the liver and enhance contact between Kupffer cells and circulating material thus favouring increased particle trapping in the liver during crisis.

Conspicuously, mRNA levels of iNOS correlate well with those of VDR in both non-vaccinated and vaccinated mice. The mRNAs of iNOS and VDR were strongly up-regulated by *P. chabaudi *infection during crisis on days 8 and 11 p.i., and vaccination largely prevented this effect. In this context, recent findings are noteworthy that VDR signalling is able to inhibit IFN-γ-induced expression of macrophage activation markers including CXCL10 [51] and mitigates production of toxic nitric oxide by inducing arginase expression which lowers the intracellular pool of the iNOS substrate arginine [52]. In non-vaccinated mice, induction of arginase is presumably only of minor importance since iNOS induction is more than 100-fold stronger than induction of arginase. The up-regulation of VDR by infection in non-vaccinated mice may thus represent an insufficient counter-regulatory response to protect liver tissue from excessive damages due to extreme up-regulation of iNOS producing high NO levels. In vaccinated mice, VDR up-regulation appears to be dispensable since there is only minor induction of iNOS. Although vaccination strongly down-regulates markers for classically activated M1 macrophages such as TNF and iNOS, it does not apparently result in differentiation of macrophages to an alternatively activated M2 phenotype, since arginase as marker for alternatively activated macrophages [53] is even down-regulated by vaccination.

The vaccination-induced attenuation in the inflammatory response of the Kupffer cells appears to have modulatory effects on hepatocyte-based reactions involved in the defence against blood stages of malaria. Indeed, data indicate that vaccination diminishes the infection-induced acute phase response with respect to the CRP- and SAA3-proteins. Also, vaccination affects the infection-induced metabolic response with respect to SULT2A1 and CYP7A1, respectively. The phase I enzyme CYP7A1 is involved in cholesterol degradation/bile acid biosynthesis and the phase II enzyme SULT2A1 in the detoxification of bile acids, the latter majorly derived from cholesterol released during destruction of pRBC. At peak parasitaemia, the vaccination-induced elevated levels of SULT2A and decreased levels of CYP7A1 may be, therefore, part of a protective response, which diminishes liver damages due to high levels of toxic bile acids.

Liver metabolism including phase I and II detoxifications are not only regulated by CAR, but also by other nuclear receptors such as FXR, PXR, and RXR. Here, it is shown that these nuclear receptors are also responding to both challenge-infection and vaccination due to cytokines released by Kupffer cells. Conspicuously, the nuclear receptors PXR, FXR, RXR, CAR, and VDR are significantly up-regulated during crisis after vaccination, while non-vaccinated mice exhibit a down-regulation. However, the results concerning CAR activation with TCPOBOP show that mRNA levels of nuclear receptors are not predictive for hepatic metabolic capacity during blood stage malaria. The expression of SULT2A and CYP7A1 is known to respond positively and negatively to CAR activation, respectively. Direct testing of CAR receptor function by treating non-vaccinated mice with TCPOBOP revealed that both *P. chabaudi *infection and vaccination altered CAR functionality on day 8 p.i. In non-vaccinated mice, Sult2a was no longer up- but even down-regulated by TCPOBOP on day 8 p.i. Vaccination prevented Sult2a down-regulation and retained its inducibility by TCPOBOP. Surprisingly, however, vaccination also abolished TCPOBOP-mediated down-regulation of Cyp7a1 on day 8 p.i. and even caused induction of Cyp7a1 by TCPOBOP. At peak parasitaemia, vaccination apparently promotes up-regulation of gene expression by CAR and, concomitantly, interferes with down-regulation. The simplest explanation for this complex response pattern is that CAR interacts with different cofactors, which determine whether it acts as a transcriptional activator or repressor. Characterization of such cofactors in the future will help to identify pathways leading to liver dysfunction in malaria and thus provide important information for prophylactic and therapeutic interventions.

## Conclusion

The improvement of hepatic trapping and metabolic capacity in protectively vaccinated mice support the previous view that the liver has an effector function against blood stage malaria – at least during acute infection. This effector function can be strengthened by vaccination due to dampening the production of the proinflammatory cytokines IL-1β, TNF, and IL-6 by Kupffer cells. Moreover, the data presented here suggest that the efficacy of a human anti-malaria vaccine can be improved by including anti-inflammatory components protecting the liver from overwhelming inflammatory responses.

## Competing interests

The authors declare that they have no competing interests.

## Authors' contributions

JK, HM, and FW designed the study. JK and FW planned and supervised all experiments and drafted the manuscript. DD and HP performed real-time RT-PCR analyses, AW did Northern blot experiments, MEK and MD did particle trapping experiments. All authors were involved in revising the manuscript and approved the final version.

## Supplementary Material

Additional file 1 **Additional figure**. Diagram showing blood glucose level, anaemia, and percentage of reticulocytes in the blood between vaccinated and non-vaccinated mice. Click here for file
