# Supplementary figures and images for: Augmented particle trapping and attenuated inflammation in the liver by protective vaccination against Plasmodium chabaudi malaria
Source: Malar J. 2009 Apr 2;8:54. doi: 10.1186/1475-2875-8-54 (PMC2679048; doi:10.1186/1475-2875-8-54)

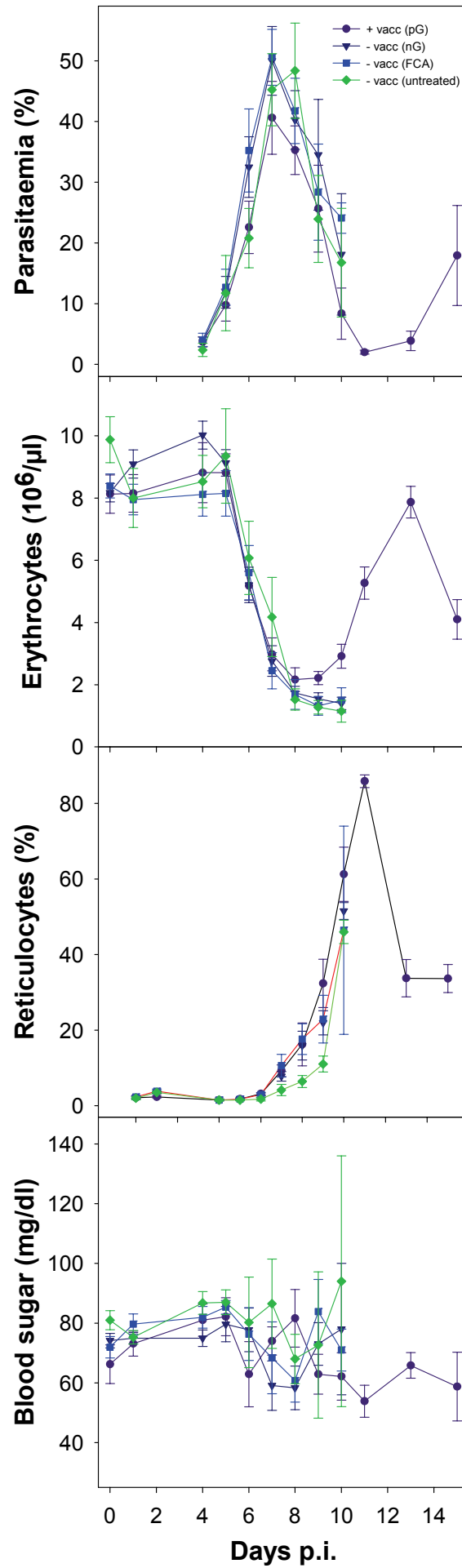

Supplement: Additional file 1 — Additional figure. Diagram showing blood glucose level, anaemia, and percentage of reticulocytes in the blood between vaccinated and non-vaccinated mice. [file 1475-2875-8-54-S1.pdf]
